# Enhanced NiFe_2_O_4_ Catalyst Performance and Stability in Anion Exchange Membrane Water Electrolysis: Influence of Iron Content and Membrane Selection

**DOI:** 10.3390/molecules30153228

**Published:** 2025-08-01

**Authors:** Khaja Wahab Ahmed, Aidan Dobson, Saeed Habibpour, Michael Fowler

**Affiliations:** Department of Chemical Engineering, University of Waterloo, Waterloo, ON N2L 3G1, Canada

**Keywords:** AEM, PEM, OER, EIS

## Abstract

Anion exchange membrane (AEM) water electrolysis is a potentially inexpensive and efficient source of hydrogen production as it uses effective low-cost catalysts. The catalytic activity and performance of nickel iron oxide (NiFeO_x_) catalysts for hydrogen production in AEM water electrolyzers were investigated. The NiFeO_x_ catalysts were synthesized with various iron content weight percentages, and at the stoichiometric ratio for nickel ferrite (NiFe_2_O_4_). The catalytic activity of NiFeO_x_ catalyst was evaluated by linear sweep voltammetry (LSV) and chronoamperometry for the oxygen evolution reaction (OER). NiFe_2_O_4_ showed the highest activity for the OER in a three-electrode system, with 320 mA cm^−2^ at 2 V in 1 M KOH solution. NiFe_2_O_4_ displayed strong stability over a 600 h period at 50 mA cm^−2^ in a three-electrode setup, with a degradation rate of 15 μV/h. In single-cell electrolysis using a X-37 T membrane, at 2.2 V in 1 M KOH, the NiFe_2_O_4_ catalyst had the highest activity of 1100 mA cm^−2^ at 45 °C, which increased with the temperature to 1503 mA cm^−2^ at 55 °C. The performance of various membranes was examined, and the highest performance of the tested membranes was determined to be that of the Fumatech FAA-3-50 and FAS-50 membranes, implying that membrane performance is strongly correlated with membrane conductivity. The obtained Nyquist plots and equivalent circuit analysis were used to determine cell resistances. It was found that ohmic resistance decreases with an increase in temperature from 45 °C to 55 °C, implying the positive effect of temperature on AEM electrolysis. The FAA-3-50 and FAS-50 membranes were determined to have lower activation and ohmic resistances, indicative of higher conductivity and faster membrane charge transfer. NiFe_2_O_4_ in an AEM water electrolyzer displayed strong stability, with a voltage degradation rate of 0.833 mV/h over the 12 h durability test.

## 1. Introduction

Several existing renewable energy sources have considerable limitations in terms of capacity, being highly conditional on specific conditions and influenced by uncontrollable factors such as the availability of sunlight and wind speeds [1]. Therefore, many such sources are unable to keep up with peak power requirements and produce excess power during low usage times. In this context, hydrogen shows considerable promise in acting as both a fuel and a storage medium for these other renewable energy sources. Hydrogen has a very high specific energy density by mass, and can produce three times as much energy as gasoline [2,3]. Furthermore, it is versatile in utilization, produces no pollutants during use, and as a gas is relatively cheap to transport [4].

There are several methods currently employed to generate hydrogen including water electrolysis, gasification, fermentation, steam reforming, photocatalytic, and water thermolysis [5]. Water electrolysis shows considerable potential for sustainable hydrogen generation among the mentioned methods. While it is more expensive and less utilized than other methods such as steam reforming, it produces no pollutants or environmentally harmful byproducts. Specifically, electrolysis based on renewable sources, such as hydroelectric or wind energy, produces at least three times less CO_2_ per kg of H_2_ when compared to steam reforming [6,7].

There are three primary methods of water electrolysis: alkaline water electrolysis (AWE), proton exchange membrane (PEM) water electrolysis, and anion exchange membrane (AEM) water electrolysis. Currently, only AWE is widespread on an industrial scale, which can primarily be attributed to the low cost of AWE systems. However, despite being the most frequently used, AWE shows several limitations such as low current densities and comparatively lower purity and pressure compared to PEM and AEM systems [8,9]. PEM systems show better performance and purity; however, the high costs associated with the development and operation of PEM electrolyzers present significant barriers, making their widespread adoption challenging. PEM water electrolyzers require expensive structural materials due to the acidic operational environment, generally consisting of titanium or other noble metals in the gas diffusion mesh and bipolar plates. Furthermore, for the same reason, noble metal catalysts are required. The scarcity of the materials used results in high operational costs that often render PEM water electrolyzers uneconomical [10,11].

AEM electrolyzers have been receiving considerable attention and have been the focus of developmental efforts due to their innate advantages over AWE and PEM systems. AEM systems utilize an anion exchange membrane (AEM) and operate in a basic solution of 0.1–1 M KOH or NaOH [12]. Operation in water is possible; however, it results in lower performance as the electrolytes in the solution increase conductivity and reduce the corrosion of the catalyst [13]. The alkaline environment allows for non-noble metal catalysts, such as transition metal oxides, as well as removing the structural material requirements found in PEM systems. This allows AEM electrolyzers to use more common and cheaper materials, typically substituting platinum group materials (PGMs) for nickel-based materials [14]. These fundamental differences allow for reduced costs when compared to PEM water electrolyzers, while displaying greater performance compared to AWE systems [15,16].

Despite their advantages over AWE and PEM systems, there are still several key challenges in utilizing AEM electrolyzers for hydrogen generation. AEM electrolyzers, despite their lower cost, show lower current density and energy efficiency when compared to PEM water electrolyzers [17]. These deficiencies can be largely attributed to high overpotentials found in oxygen evolution reaction (OER) catalysts. As such, one primary focus for development in AEM systems is the development of low overpotential catalysts. Noble metal oxides have been found to be effective for this purpose. However, they are scarce and expensive to use. Perovskite- and Chalcogenide-based catalysts with bifunctional catalytic activity for the OER and HER have also been an area of research in the non-PGM catalyst search [18,19,20]. A chemically bifunctional catalyst, LaCoO_3_-MoS_2_, has been shown as a noble-metal-free electrocatalyst for alkaline water splitting, achieving efficient hydrogen evolution reaction (HER) and OER performance [21]. Spinel-structured first-row transition metal oxides have received the most attention due to their efficiency as electrocatalysts and their relative abundance [22,23,24]. NiFe_2_O_4_-based catalysts show promise and potential for this application. One reason is due to the polyvalent redox pairs present (Ni^2+^/Ni^3+^ and Fe^2+^/Fe^3+^), which can act as redox active sites for oxygen evolution [25]. Additionally, they possess high stability as well as corrosion resistance, which helps mediate a major concern in the long-term operation of AEM electrolyzers [22]. Furthermore, they are abundant and high in reserve, resulting in low costs, especially when compared to noble metal-based catalysts [25].

NiFe_2_O_4_-based catalysts have shown high performance in the literature for OER and AEM applications. NiFe_2_O_4_ nanoparticles, sized at 10 nm, showed high activity in the OER, achieving a current density of 10 mA cm^−2^ at an overpotential of 290 mV, with a Tafel slope of 42 mV dec^−1^ [22]. Furthermore, NiFe_2_O_4_ nanorods have also shown excellent performance, with mesoporous NiFe_2_O_4_ nanorod catalysts reaching 10 mA cm^−2^ at an overpotential of 342 mV, with a Tafel slope of 44 mV dec^−1^ [23]. Furthermore, doping or mixing NiFe_2_O_4_ with various materials has shown an increase in performance. NiO/NiFe_2_O_4_ biphasic nanorods achieve 10 mA cm^−2^ at a reduced overpotential of 302 mV, with a slightly lower Tafel slope of 42 mV dec^−1^, and an increased specific surface area when compared to standard NiFe_2_O_4_ nanorods [24]. A NiFe_2_O_4_@Co_3_O_4_ heterostructure catalyst achieved 10 mA cm^−2^ at an overpotential of 251 mV with a low Tafel slope of 36 mV dec^−1^ [26]. The electrocatalytic performance of pentlandite-like Fe_x_Ni_9−x_S_8_ nanoparticles for the oxygen evolution reaction has been systematically studied, demonstrating improved activity with increasing nickel content [27]. For AEM water electrolysis, NiFe_2_O_4_ catalysts have been studied and reported to have good activity.

In this study, NiFeO_x_ and NiFe_2_O_4_ catalysts were synthesized; in NiFeO_x_ catalysts, the Fe content was changed from 5 to 12.5 wt% of total metal content. The OER was performed in three-electrode systems, and complete water electrolysis was performed using an AEM electrolyzer. Electrochemical impedance spectroscopy (EIS) was performed to evaluate different resistances due to the electrolyzer components. Long-term and short-term durability testing was also performed using NiFe_2_O_4_ at the anode. In addition, several anion exchange membranes (AEMs) were tested, and their performance was compared for AEM water electrolysis.

## 2. Results

**Figure 1:**
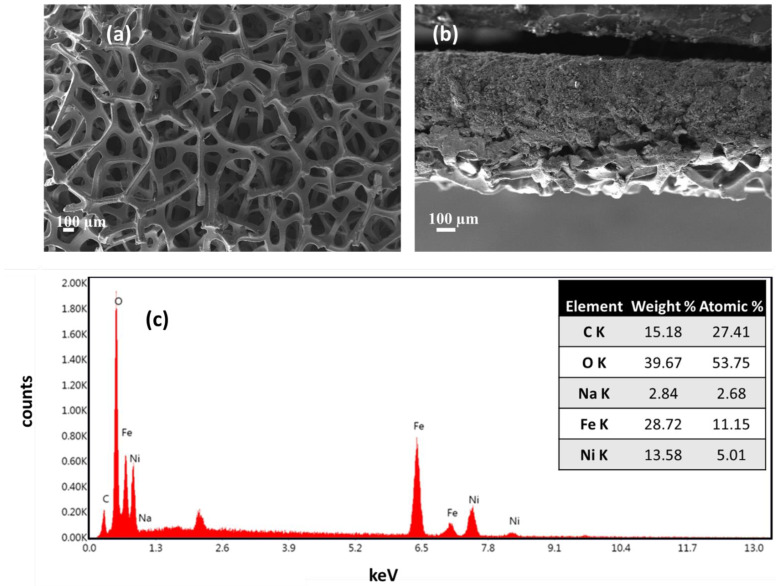
(**a**) SEM of nickel foam anode GDL; (**b**) cross-section of NiFe_2_O_4_ catalyst-coated nickel foam anode; (**c**) EDS of NiFe_2_O_4_ catalyst, which shows the ratio of Ni to Fe of about 1:2.

SEM and EDS analyses were carried out using the Leo 1550 SEM (Zeiss, Dublin, CA USA). Figure 1a shows the morphology of the nickel foam anode GDL. Nickel foam had a pore size in the range of 100 to 500 μm, which provides easier access to water and oxygen at the anode to transport. The cross-sectional image shows the total width of the catalyst-coated nickel foam, which had a thickness of 740 μm, as shown in Figure 1b. The NiFe_2_O_4_ catalyst layer was dense, and catalyst particles were distributed within the matrix of the nickel foam. Figure S1 in the Supplementary Material shows the schematic of the membrane electrode assembly, where nickel foam was used as a gas diffusion layer at the anode, and the NiFeO_x_ or NiFe_2_O_4_ catalyst layer was used on top of it, while at the cathode side, a carbon paper GDL was used with a Pt/C catalyst. Figure 1c shows the EDS of NiFe_2_O_4_, which clearly shows the Ni-to-Fe ratio of approximately 1:2. The EDS results of other catalysts are presented in Figures S2–S4 in the Supplementary Material. In all cases, the catalyst composition was confirmed from the EDS analysis.

Catalysts were analyzed by XRD (Rigaku Miniflex 600, Tokyo, Japan) using Cu-Kα radiation (λ = 1.54184 Å).

**Figure 2:**
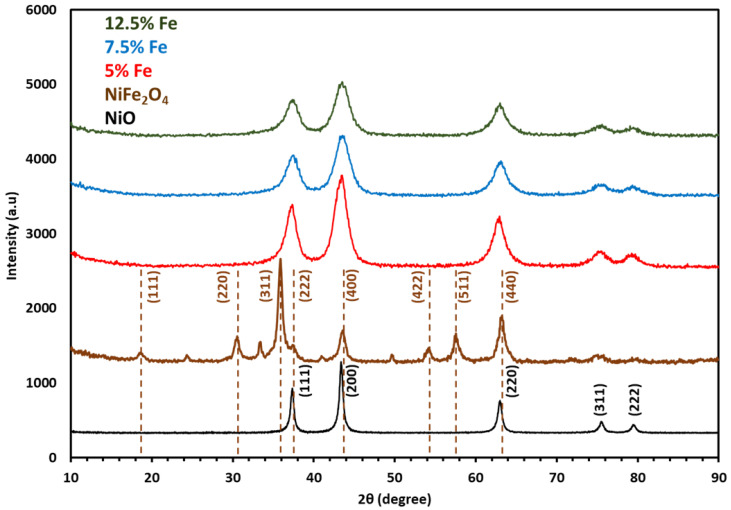
XRD of NiFeO_x_ and NiFe_2_O_4_ catalysts.

NiFeO_x_ samples with varying Fe concentrations of 5%, 7.5%, and 12.5% exhibit the NiO phase, characterized by five distinctive peaks at 2θ values of 37.36, 43.36, 63, 75.54, and 79.56. These peaks are assigned to the crystallographic planes (hkl) of (111), (200), (220), (311), and (222), respectively. The diffraction peaks have been matched and indexed to NiO using the JCPDS card number 78-0643, as depicted in Figure 2.

The NiFe_2_O_4_ catalyst showed the typical diffraction pattern of NiFe_2_O_4_, with the main peak centered at 2θ 18.6, 30.5, 35.5, 37.5, 43.6, 54.3, 57.5 and 63.2, which are assigned to (111), (220), (311), (222), (400), (422), (511), and (440), respectively. The XRD data includes dashed lines representing the JCPDS standard for NiFe_2_O_4_. The JCPDS card number 22-1086 was used to match the diffraction lines of NiFe_2_O_4_.

### 2.1. Oxygen Evolution Reaction

**Figure 3:**
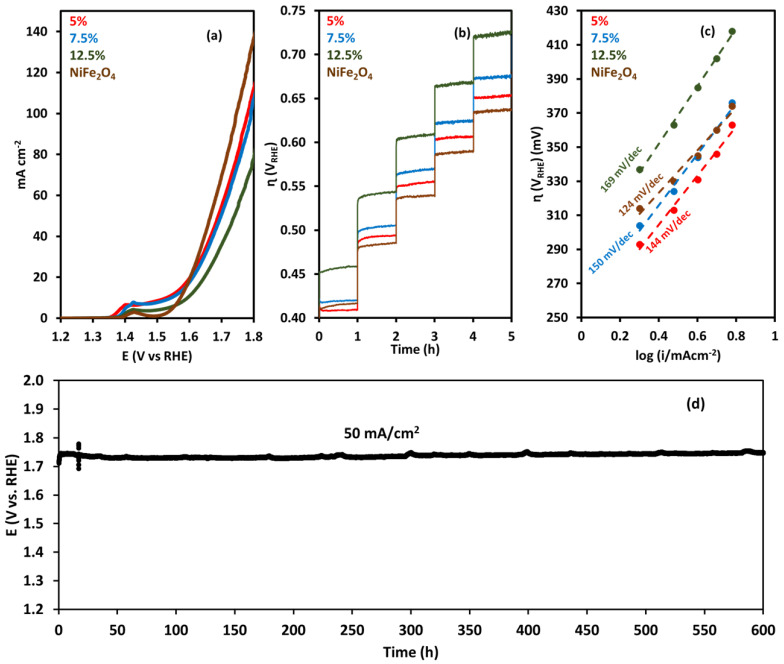
OER at room temperature in 1 M KOH with NiFe_2_O_4_ and NiFeO_x_ catalysts: (**a**) LSV (85% iR-corrected); (**b**) chronoamperometry in current density range of 10 to 100 mA/cm^2^; (**c**) Tafel plots for OER; (**d**) catalyst Catalyst durability test for 600 h at current density of 50 mA/cm^2^ using NiFe_2_O_4_ catalyst at anode.

To evaluate the catalytic activity of the NiFeO_x_ catalyst, linear sweep voltammetry was performed in 1 M KOH. NiFe_2_O_4_ showed the highest catalytic activity with a current density of 135 mA cm^−2^ at 1.8 V (Figure 3a). Figure 3b shows chronopotentiometry at current densities of 10 to 50 mA cm^−2^. At each voltage, the operation was performed for 1 h, which shows the short-term stability of the catalyst at these conditions. The lowest overpotential was 409 mV and 416 mV for the NiFeO_x_ (5%) and NiFe_2_O_4_ catalysts, respectively. The highest overpotential was for NiFeO_x_ (12.5%), which was 457 mV, followed by the overpotential of 419 mV for NiFeO_x_ (7.5%). The catalyst performance was stable at a higher current density of 50 mA cm^−2^. These results show that the catalysts are stable under high-current-density operation and well suited for AEM electrolysis. Tafel plots, as depicted in Figure 3c, were used to compare the efficiency of different catalysts. These plots represent the correlation between overpotential *η* and current density (*i*) on a logarithmic scale. The Tafel slope (*b*), derived from the linear segment of the plot, is indicative of the catalyst’s activity. NiFe_2_O_4_ has a Tafel slope of 124 mV/dec and NiFeO_x_ (12.5%) has a Tafel slope of 169 mV/dec. The Tafel slopes of NiFeO_x_ (5%) and NiFeO_x_ (7.5%) were 144 mV/dec and 150 mV/dec, respectively, which are in the same range. These results showed that the NiFe_2_O_4_ catalyst is a relatively more active catalyst among the series of catalysts. The value of the Tafel slope helps identify the rate-determining step (RDS) in the reaction mechanism. A Tafel slope of ~120 mV/dec typically indicates that the first electron transfer step (M + H_2_O → M–OH* + H^+^ + e^−^) is the rate-determining step (RDS). A slope near 60 mV/dec suggests that a chemical step following an electron transfer (M–OH* → M–O* or M–O* + H_2_O → M–OOH* + H^+^ + e^−^) is rate-limiting. A 30 mV/dec slope suggests that the surface coverage of intermediates is high, and the chemical recombination step (M–O → O_2_) is the RDS. In our case for all the catalysts, the Tafel slope is higher than 120 mV/dec, which shows that the adsorption of OH^−^ on the catalyst is the rate-determining step.

Figure S5 in Supplementary Information shows the EIS analysis at 1.7 V (vs. RHE) at 25 °C in 1 M KOH NiFe_2_O_4_ catalysts show a smaller semicircle compared to the other catalysts. The diameter of the semicircle in the Nyquist plot shows the activation resistance. This shows that the NiFe_2_O_4_ catalyst is more active in charge transfer with surface-adsorbed intermediates compared to other catalysts.

To test the activity and stability of NiFe_2_O_4_ catalysts for the OER, a durability test was performed for 600 h as shown in Figure 3d. The test was performed at a current density of 50 mA/cm^2^. The initial voltage was 1.739 V (vs. RHE), and during the 600 h operation, the voltage increased to 1.748 V. The degradation rate or the voltage increase rate was 15 μV/h during the 600 h OER test.

**Figure 4:**
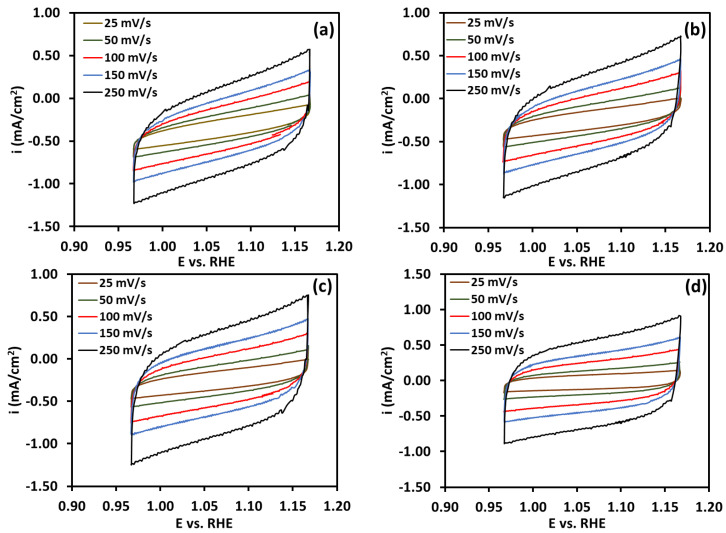
Cyclic voltammetry at different scan rates; the analysis was performed in non-Faradic region. (**a**) NiFeO_x_ (5% Fe); (**b**) NiFeO_x_ (7.5% Fe); (**c**) NiFeO_x_ (12.5% Fe); (**d**) NiFe_2_O_4_.

**Figure 5:**
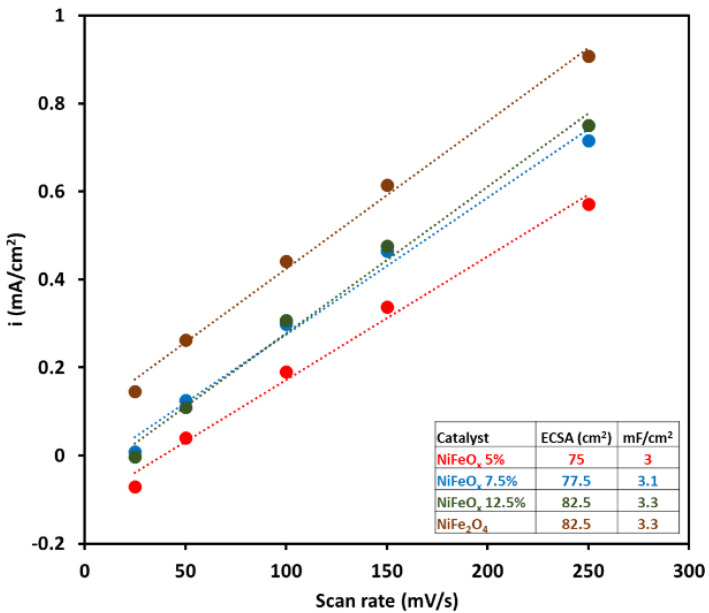
Double-layer capacitance (C_dl_) plot of scan rate vs. current density for NiFeO_x_ and NiFe_2_O_4_ catalysts.

Figure 4a–d shows the CV plot; the scan rate ranged from 25 to 250 mV s^−1^, and the voltage ranged from 0.967 V to 1.167 V (non-Faradaic region for water electrolysis). C_dl_, the double-layer capacitance, was calculated from the slope of the scan rate vs. current density plot in Figure 5. The electrochemical surface area (ECSA) was 75 cm^2^, 77.5 cm^2^, 82.5 cm^2^, and 82.5 cm^2^ for the NiFeO_x_ (5% Fe), NiFeO_x_ (7.5%), NiFeO_x_ (12.5% Fe), and NiFe_2_O_4_ catalysts, respectively.

### 2.2. AEM Electrolyzer Tests with NiFeO_x_ and NiFe_2_O_4_ Catalysts

**Figure 6:**
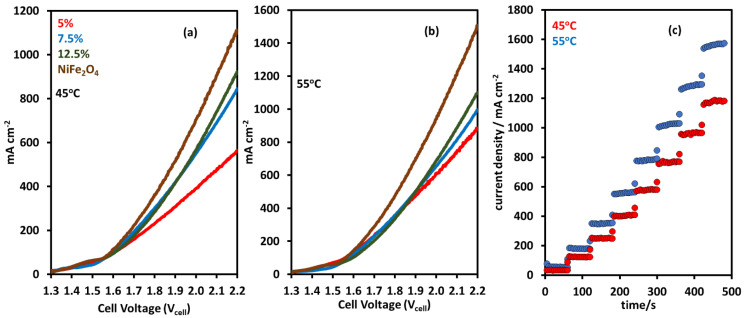
Linear sweep voltammetry in a temperature range of (**a**) 45 °C and (**b**) 55 °C. NiFeO_x_ and NiFe_2_O_4_ catalysts were used at the anode. X-37-50 T membrane was used for AEM electrolysis; (**c**) chronoamperometry in a voltage range of 1.5 to 2.3 V at 45 °C and 55 °C, with NiFe_2_O_4_ catalysts.

Single-cell tests were performed with the NiFeO_x_ catalyst at the anode and Pt/C at the cathode, using Ni foam and carbon paper GDLs, respectively, and a dioxide membrane (X-37-50 grade T). The AEM X-37-50 T from Sustainion^®^ exhibits high conductivity and stability in AEM water electrolysis [28,29]. The membrane comprises co-polymers of styrene and vinyl benzyl chloride supported by polytetrafluoroethylene (PTFE), incorporating imidazolium and pyridinium groups for ion exchange. Figure 6a shows polarization curves using NiFe_2_O_4_ catalysts and NiFeO_x_ catalysts at 45 °C. NiFe_2_O_4_ showed the highest activity of 1100 mA cm^−2^ at 2.2 V. Other catalysts also showed good activity for AEM electrolysis; at the same voltage, the catalyst with 12.5 wt% Fe had a current density of 921 mA cm^−2^. The current density decreased to 842 mA cm^−2^ and 561 mA cm^−2^ for catalysts with Fe contents of 7.5 wt% and 5 wt%. Figure 6b shows the polarization curves at 55 °C; for all the catalysts, the activity increased with the increase in temperature. The same trend emerges in the activity of the catalysts during the 45 °C electrolysis operation. NiFe_2_O_4_ shows the highest current density of 1503 mA cm^−2^, which is an increase of 36%. Current densities were 1098 mA cm^−2^, 994 mA cm^−2^, and 885 mA cm^−2^ for the catalysts with 12.5 wt%, 7.5 wt%, and 5 wt% Fe content.

Temperature is an important factor in the performance of an AEM electrolyzer, which must be considered during catalyst performance analysis. Increased temperature results in lower overpotentials for any given current density, as well as an increase in the efficiency of the electrolyzer [30,31,32]. The improved reaction kinetics play a large role in this, as the electrochemical reaction kinetics increase with temperature [33]. Furthermore, kinetics improve due to the reduced energy requirement for splitting water molecules, as there is additional energy present within the system. Increased ionic conductivity at higher temperatures also plays a large role in increasing performance due to the increase in mobility of OH^−^ ions, increasing their availability for the redox reaction [34].

The NiFe_2_O_4_ catalyst shows good activity for AEM water electrolysis in different operational conditions as reported in the literature. A current density of 1000 mA cm^−2^ was achieved at 1.85 V for AEM electrolysis using NiFe_2_O_4_ as the anode catalyst and Raney Nickel as the cathode catalyst in a 1 M KOH solution, with a Sustainion X-37-50 T membrane at 60 °C [35]. Additionally, a system using NiFe_2_O_4_ at the anode and 30 wt% PtNi/ECS (Engineered Catalyst Support, carbon) at the cathode had a current density of 1000 mA cm^−2^ in 60 °C 0.1 M NaOH, using a GT72-10 with PTFE reinforcement with a thickness of 30 μm [36]. Similar activity was reported in another study using NiFe_2_O_4_ at the anode and NiFeCo at the cathode in a 1 M KOH solution at 60 °C, with a current density of 900 mA cm^−2^ reported at 2.0 V using a FAS-50 membrane [37].

Chronoamperometry (CA) was used to measure the activity of the catalyst at a constant voltage. Figure 6c shows the chronoamperometry at 45 °C and 55 °C; the voltage ranged from 1.5 V to 2.2 V in time steps of 60 s. Figure 6c shows the CA results on the NiFe_2_O_4_ catalyst; the current density increased from 32 mA cm^−2^ to 1179 mA cm^−2^ when the voltage was increased in steps from 1.5 V to 2.2 V at 45 °C. The activity also increased when the operation was performed at a higher temperature of 55 °C; the highest current density at 2.2 V was 1568 mA cm^−2^.

### 2.3. Effect of Anion Exchange Membranes on Electrolyzer Performance

Anion exchange membranes have a significant impact on the performance of AEM water electrolyzers, due to their critical role in their operation. The specific membrane choice can also play a large role in determining the electrolyzer performance. Several characteristics determine the performance of a specific membrane; however, specifically predicting and ranking these membranes requires experimental results and is often system-specific [38]. Maximizing ionic conductivity and stability while reducing specific resistance is essential to increase performance and viability. Mechanical stability frequently comes at the cost of reducing ionic conductivity and vice versa [39]. This is because thinner membranes frequently exhibit better OH^−^ transfer and lower mass transport resistance [40]. The membrane manufacturer Fumatech offers a wide range of membranes with different characteristics and performance. Out of these membranes, the following are of relevance to this study: FAA-3-50, FAS-50, FAA-3-PK-75, and FAB-PK-130.

The Fumasep FAA-3-50 is a membrane based on a brominated polysulfone backbone and uses quaternary ammonium (QA) sidechain groups [41]. All mentioned Fumatech membranes come with a Br^−^ counter ion; however, this can be altered as specified in their technical data sheet. FAA-3-50 is comparatively thin, at 45–55 µm. It has a very low specific area resistance at 0.6–1.5 Ω cm^2^ and has a high specific conductivity of 3–8 mS cm^−1^ [42]. Table 1 shows the specifications of Fumasep membranes from the membrane supplier; the conductivities and area-specific resistance of the membranes are reported in Cl^−^ form. The Fumasep FAS-50 membrane is composed of propriety hydrocarbon resin, and its composition is not available. This membrane is similar to FAA-3-50 in terms of performance specifications, with a thickness of 45–55 µm, specific area resistance of 0.6–1.5 Ω cm^2^, and a high specific conductivity of 3–8 mS cm^−1^ [43]. FAA-3-PK-75 is also based on a polysulfone backbone with the same QA sidechains, alongside a poly-ketone (PK) reinforcement, and has a thickness of 70–80 µm [44]. This thickness, alongside its high specific area resistance of 1.2–2.0 Ω cm^2^, results in a lower specific conductivity of 4.5–6.5 mS cm^−1^ [45]. The final membrane examined is FAB-PK-130, composed of PK-reinforced hydrocarbon resin [46]. This membrane was designed to have high mechanical stability and stability in acidic and basic environments. FAB-PK-130 is a much thicker membrane of 110–140 µm and, as a result, has a very high specific area resistance of 5.0–9.0 Ω cm^2^. This results in low specific conductivities of 1.0–2.5 mS cm^−1^. The conductivities of the Fumasep FAA-3-50 and X-37-50 membranes in KOH have been reported to be 40 and 80 mS cm^−1^, respectively, considerably higher than the membrane’s conductivity in chloride form [38].

**Figure 7:**
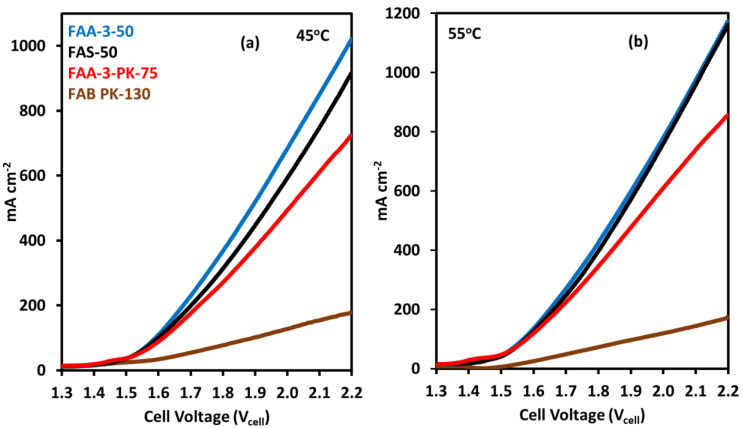
AEM electrolysis comparison using FAA-3-50, FAS-50, FAA-3-PK-75, and FAB PK-130 membranes at (**a**) 45 °C and (**b**) 55 °C, with NiFe_2_O_4_ catalyst at anode and 1 M KOH as electrolyte.

For the comparison of electrolyzer activity with different AEMs, commercially available Fumasep membranes FAA-3-50, FAS-50, FAA-3-PK-75, and FAB PK-130 were used. AEM electrolysis was performed at 45 and 55 °C with 1 M KOH, using a NiFe_2_O_4_ catalyst at the anode and Pt/C catalyst at the cathode. FAA-3-50 MEA showed the highest current density of 1020 mA cm^−2^ at 2.2 V, as shown in Figure 7a. FAS-50 MEA showed similar activity, with a current density of 915 mA cm^−2^. The activity of FAA-3-PK-75 was also high, with a current density of 723 mA cm^−2^, but compared to other membranes, the activity was slightly lower. The lowest activity was that of the FAB PK-130 membrane, with a current density of 178 mA cm^−2^.

At 55 °C, the current density increased for each membrane but the trend in activity was the same as at 45 °C (Figure 7b). The MEAs with FAA-3-50 and FAS-50 achieve current densities of 1174 mA cm^−2^ and 1161 mA cm^−2^ at 2.2 V. For the FAA-3-PK-75 membrane, current density increased to 857 mA cm^−2^, which is an about 18% increase compared to the 45 °C operation. The current density did not increase for the FAB PK-130 membrane with the increase in temperature, which means that this membrane is the least active for AEM electrolysis compared to the other membranes used in this study.

The activities of the different membranes follow the trends predicted by their specific conductivity. The overall activity of an AEM electrolyzer is strongly influenced by the conductivity of the membrane, as it allows easier passage of ions with minimal resistance, resulting in greater current densities. The higher specific conductivity of FAA-3-50 and FAS-50 resulted in higher activity when compared to FAA-3-PK-75 and FAB PK-130. This is corroborated by the results, where the FAA-3-50 and FAS-50 membranes show higher activity at 2.2 V. The comparatively lower conductivity of FAA-3-PK-75, at 1.5 mS cm^−1^, which is approximately 19% lower compared to FAA-3-50 and FAS-50, resulted in notably reduced activity. The current density of 723 mA cm^−2^ achieved by the FAA-3-PK-75 membrane is lower than the current densities of FAA-3-50 and FAS-50 at 1020 mA cm^−2^ and 915 mA cm^−2^ respectively, showing an approximate decrease in activity of 29% and 21%. The reduced conductivity of FAB PK-130 resulted in a decrease in activity compared to all membranes.

Fumatech FAS-50, using a NiFe_2_O_4_ anode catalyst and a NiFeCo cathode in a 1 M KOH solution at 60 °C, achieved a current density of 250 mA cm^−2^ at 1.8 V and 900 mA cm^−2^ at 2 V [47]. The same catalysts and solutions achieved 240 mA cm^−2^ at 1.8 V using a FAA-3-50 membrane; current densities at 2 V were not provided. This is similar to the trends observed in this study, but with higher current densities and comparable results between FAS-50 and FAA-3-50.

#### 2.3.1. EIS Effect of Voltage and Temperature

EIS analysis was performed on the AEM water electrolysis operation at a constant voltage; the analysis was performed at a temperature range of 45 °C and 55 °C. EIS analysis was performed to analyze the impedance associated with the electrolysis process [48]. This analysis can provide information on the kinetic and mass transport processes that take place at the cathode and anode. With this analysis, the resistance of the electrolyte and membrane electrode assembly can also be evaluated [49].

**Figure 8:**
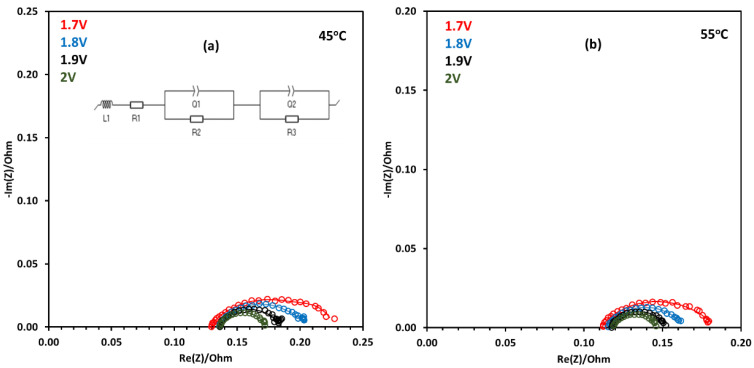
Nyquist plots for NiFe_2_O_4_ catalyst using FAA-3-50 membrane for water electrolysis at (**a**) 45 °C and (**b**) 55 °C.

Figure 8a shows the Nyquist plot for the EIS analysis performed at 45 °C. AEM electrolysis was carried out using a NiFe_2_O_4_ catalyst at the anode and Pt/C at the cathode. At 2 V, the diameter of the semicircle in the Nyquist plot is smaller compared to that at 1.7 V operation, which shows that it has lower activation resistance compared to AEM electrolysis at low voltages. In the Nyquist plot, the (high-frequency region) x-intercept shows the ohmic resistance, and the second x-intercept shows the total resistance, which has contributions from ohmic resistance, activation resistance, and mass transport resistance [50]. Figure 8b shows a similar trend, but the ohmic and activation resistances are relatively lower at 55 °C.

The Nyquist plot obtained from the EIS analysis was fitted to the equivalent circuit model using the randomized simplex method, and the results are presented in Table 2. The software EC-Lab (version V11.36) was used to perform the equivalent circuit fitting. An electrical circuit is shown in Figure 8a, which represents the components of the AEM electrolyzer. R1 is the resistance due to the membrane or electrolyte, while R2 and R3 represent activation resistances at the electrodes for the OER and HER. Q2 and Q3 represent constant phase elements (CPEs) at the electrodes. L is the inductance due to the wires used to connect the AEM electrolyzer [51].

Table 2 shows that with the increase in temperature of the AEM electrolyzer, there is a decrease in ohmic resistance, which can enhance the overall efficiency of the electrolysis process. The ohmic resistance decreased due to the improved conductivity of hydroxyl ions with the increase in temperature [52]. Table 2 shows that as the temperature rose from 45 to 55 °C, the activation resistance at both the anode and cathode decreased, from 0.101 Ω to 0.075 Ω at 2 V and from 0.055 Ω to 0.031 Ω at 2 V. With the increase in temperature, the reaction kinetics of AEM electrolysis became faster as it overcame the kinetic barrier of the reactions at both the cathode and anode [53,54]. At each voltage, the operation at 55 °C shows lower ohmic resistance compared to the 45 °C electrolysis operation.

The effect of temperature on the resistance of an AEM electrolyzer can be found in some of the AEM electrolysis studies. One study utilizing a polybenzimidazole (PBI) AEM and NiFeO_x_ and NiFeCo catalysts for the anode and cathode, respectively, in 1M KOH, found that at 1.9 V, the ohmic resistance decreased from 0.0178 Ω cm^−2^ at 30 °C to 0.0156 Ω cm^−2^ at 60 °C [31]. In the same study, the activation resistance lowered with an increase in temperature, decreasing from 0.023 Ω cm^−2^ at 30 °C to 0.0114 Ω cm^−2^. Two other studies, one utilizing Sustainion X37-50 and a NiFeCo cathode catalyst and the other FAA3-50 with a 40% Pt/C cathode catalyst, both in 1 M KOH, observed a decrease in both ohmic and activation resistance as the temperature increased from 30 °C to 60 °C [37,55].

#### 2.3.2. EIS with Different Anion Exchange Membranes

**Figure 9:**
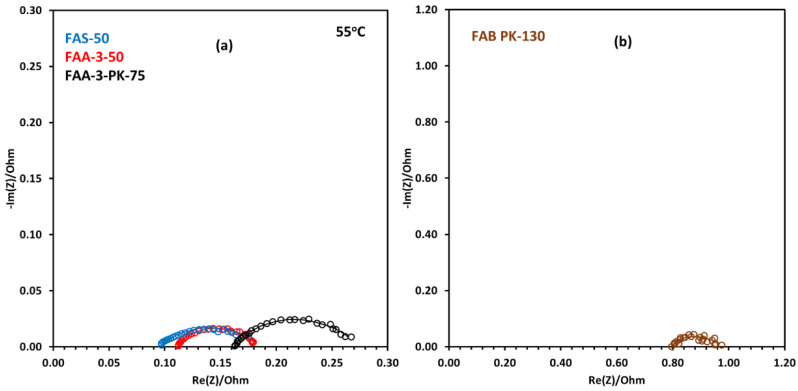
Nyquist plots for AEM electrolysis operation at 1.7 V with NiFe_2_O_4_ catalyst at 55 °C with (**a**) FAS-50, FAA-3-50 and FAA-3-PK-75 membranes and (**b**) FAB-PK-130 membrane.

Figure 9a,b show Nyquist plots at 55 °C for different membrane electrode assemblies at 1.7 V. The results of equivalent circuit fittings are presented in Table 3. The FAA-3-50 membrane showed the lowest activation resistance of 0.0756 Ω followed by FAS-50 with 0.0918 Ω. As for the other two membranes, FAA-3-PK-75 had an activation resistance of 0.113 Ω, and FAB-PK-130, which had very low electrolysis performance, had a very high activation resistance of 0.199 Ω. Similar trends were seen in ohmic resistance, with the lowest values for FAS-50 at 0.092 Ω and FAA-3-50 at 0.108 Ω. For AEM electrolysis, these membranes showed a comparable performance, which may be due to similar resistances offered during the operation. These results show that FAA-3-50 and FAS-50 had higher conductivities than the other AEMs under similar operating conditions. They also show that the membrane charge transfer between the membrane and catalyst layer is faster in these membranes compared to the low-performing membranes.

In our previous study using a Sustainion X-37-50 T membrane with a NiFeCoO_x_ anode catalyst and a Pt/C cathode catalyst in 1 M KOH, at 2 V and at 70 °C, resistances of 0.064 Ω and 0.037 Ω were obtained for ohmic and activation resistance, respectively [56]. In the same study, using FAA-3-50 in an identical cell configuration resulted in resistances of 0.12 Ω and 0.054 Ω for ohmic and activation resistance, respectively. This trend continued in another study, where X-37-50 displayed a considerably lower resistance, possessing an activation resistance of 0.034 Ω compared to the 0.042 Ω of FAS-50, 0.056 Ω of the PiperION, and 0.070 Ω of FAA-3-50 at 50 °C in 1 M KOH using a NiFe-based catalyst at the anode and a 20 wt% Pt/C cathode catalyst [57].

### 2.4. Durability Test

**Figure 10:**
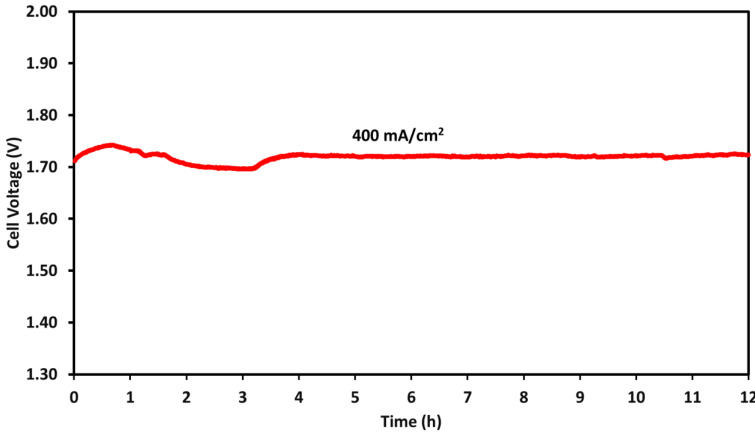
AEM electrolyzer durability test at 400 mA/cm^2^ for 12 h in 1 M KOH using NiFe_2_O_4_ catalyst at the anode; electrolyzer was operated at 55 °C using X-37-50 grade T membrane.

A durability test was performed for the AEM water electrolyzer using a NiFe_2_O_4_ catalyst at the anode. The electrolyzer was operated at 400 mA/cm^2^ for 12 h at 55 °C. Under these operating conditions, the cell efficiency was 72%, which was determined using Equation (1). A degradation rate of 0.833 mV/h was observed during the test, and the initial voltage was 1.71 V, which, after 12 h of continuous operation, increased to 1.72 V, as shown in Figure 10. The Faradic efficiency was 83%, calculated by dividing the amount of hydrogen obtained from the electrolyzer by the theoretical amount of hydrogen under these conditions. During stability testing, the electrolyzer’s performance remained stable.

The durability of AEMs affects the long-term testing of the electrolyzer operation, and commercial membranes have been reported to show losses in performance in long-term testing experiments in alkaline medium [58]. There are several key degradation pathways that are common, including various S_N_2 substitutions (benzyl, methyl), ring opening, nucleophilic substitution, dehydroflourination of the backbone, and nucleophilic addition and displacement, where specific mechanisms and the possibility of occurrence are determined by the composition of both the functional groups and backbone, as well as testing conditions [59,60]. The most commonly used functional groups, quaternary ammonium groups, are particularly susceptible to Hofmann elimination and nucleophilic substitution, which result in the degradation of cation groups and the formation of amines and olefins and amines and alcohols, respectively [61,62]. Another mechanism seen in various tetraalkylammonium hydroxides is chemical rearrangement caused by ylide formation [63,64,65]. Polymer backbones containing phenyl groups can experience degradation due to the oxidation of phenyl groups at OER potentials, leading to the formation of acidic phenols that can decrease solution pH and reduce conductivity [65,66]. A study by Arges et al. found that polysulfone-based backbones can become brittle and therefore mechanically unstable in alkaline solutions due to hydrolysis at both the ether and quaternary carbon groups when cation groups are attached to the benzyl position of the diphenyl propane group [63].

The deactivation of the catalysts can also cause voltage increases, which can occur due to a variety of factors. Changes in morphology are one such factor that has a significant impact on performance, where changes in structure can result in poor contact area between the catalyst and ionomer and reduce catalyst utilization [67]. Catalyst dissolution is another significant contributing factor in the deactivation of catalysts, where the catalysts dissolve into the electrolyte solution; this effect is typically intensified or promoted with electrolyzer operation as opposed to innately when in the electrolyte [68]. Passivation is a frequently seen deactivation method, wherein an insulating layer is formed between the catalyst and electrode, which inhibits the flow of electrons and therefore decreases cell performance [69,70]. In general, these factors typically depend on the specific catalyst used, and many such mechanisms are present at once, increasing and contributing to overall degradation [71].

## 3. Experimental

### 3.1. Synthesis of NiFeO_x_ Catalyst

The catalysts were synthesized by the coprecipitation method at room temperature, using nickel nitrate hexahydrate (Ni(NO_3_)_2_·6H_2_O) and iron nitrate nonahydrate (Fe(NO_3_)_3_·9H_2_O) as precursors for nickel and iron, respectively. The Fe content varied from 5 wt% to 12.5 wt% for NiFeOx catalysts, and for NiFe_2_O_4_ catalysts, the Ni-to-Fe ratio was kept at 1:2. For 2 g of total metal content, the following precursor quantities were used: 9.411 g of Ni(NO_3_)_2_·6H_2_O and 0.722 g of Fe(NO_3_)_3_·9H_2_O for the 5 wt% Fe catalyst (yielding 1.900 g Ni and 0.100 g Fe); 9.158 g of nickel precursor and 1.084 g of iron precursor for 7.5 wt% Fe (1.850 g Ni and 0.150 g Fe); and 8.687 g of Ni(NO_3_)_2_·6H_2_O and 1.805 g of Fe(NO_3_)_3_·9H_2_O for the 12.5 wt% Fe formulation (1.750 g Ni and 0.250 g Fe). For NiFe_2_O_4_, 3.301 g of Ni(NO_3_)_2_·6H_2_O and 9.640 g of Fe(NO_3_)_3_·9H_2_O were used to yield 0.667 g Ni and 1.333 g Fe.

Both precursors were dissolved in deionized water at room temperature. Further, 1 M NaOH was used for precipitation and was added drop by drop until complete precipitation. The precipitate was then washed with water to remove excess NaOH and then centrifuged to collect the solid catalyst. The catalyst was then dried in an oven at 100 °C overnight to remove water. For both NiFeO_x_ and NiFe_2_O_4_ catalysts, calcination was performed at 300 °C for 5 h.

### 3.2. Catalyst Testing in Three-Electrode System

Linear sweep voltammetry (LSV) and cyclic voltammetry (CV) were performed for OER and ECSA measurement using a potentiostat (VMP-3, Biologic, Seyssinet-Pariset, France). The OER three-electrode system consists of a graphite counter electrode, a standard calomel electrode (SCE) as a reference electrode, and a working electrode consisting of nickel foam with an area of 1 cm^2^ coated with the NiFeO_x_ or NiFe_2_O_4_ catalyst. To fabricate the catalyst-coated electrode, catalyst ink was prepared by mixing the catalyst with a 5 wt% Nafion solution and ethanol. The thick catalyst slurry was then pasted on top of the nickel foam. The catalyst loading on nickel foam anode was 5 mg cm^−2^. LSV was performed with a scan rate of 5 mV s^−1^ and an iR correction of 85%. The voltages were converted to reversible hydrogen electrode (RHE) potential using the Nernst equation.

The NiFe_2_O_4_ catalyst’s long-term durability test for the OER was performed using a constant current density of 50 mA cm^−2^ over a time period of 600 h. The electrochemical surface area (ESCA) was estimated by dividing the double-layer capacitance (C_dl_), obtained from cyclic voltammetry in 1M KOH at different scan rates in the non-Faradic region, by the smooth-plane capacitance (C_s_) of a flat metal surface (40 μF/cm^2^). This ratio reflects the available surface area for electrochemical reactions, considering deviations from ideal behavior.

### 3.3. Preparation of Gas Diffusion Electrode

To a fabricate gas diffusion electrode (GDE) for the anode, a catalyst slurry was prepared using the catalyst, water, isopropanol, and Nafion solution (Nafion™ 1100W from Sigma Aldrich, St. Louis, MO, USA). The mixture was sonicated for 30 min to achieve a consistent composition. The catalyst slurry was then applied on the nickel foam (thickness of 1700 μm). Hot pressing was performed after catalyst coating to obtain a uniform surface and to achieve the desired thickness of the GDE. The Nafion content was 10 wt% of the total catalyst weight, and the catalyst loading was 25 mg cm^−2^. For the cathode, Pt/C(40 wt%, HISPEC 4000, Johnson Matthey, London, UK) was used, with a loading of 1 mg cm^−2^. The catalyst ink comprised a Pt/C catalyst, isopropanol, water, and Nafion. The Nafion was 20 wt% of the catalyst weight, and the cathode GDE was formed by air spraying the ink on carbon paper (Sigracet 29BC, Meitingen, Germany). The total active area of the electrodes was 3.24 cm^2^ at the cathode and anode, with the dimensions of 1.8 cm × 1.8 cm. The membrane electrode assembly was formed by combining the anode GDE, AEM, and cathode GDE.

### 3.4. AEM Electrolyzer Single-Cell Performance Test

For electrolyzer performance testing, a potentiostat connected to a 100 A booster (VMP3B-100, Seyssinet-Pariset, France) was used. LSV with a scan rate of 5 mV s^−1^ was used for AEM electrolysis. The electrolysis operation was also performed using chronoamperometry. The electrolyzer configuration was described in our earlier studies [56,72]. Briefly, a 1 M KOH electrolyte was circulated with a flow rate of 90 mL/min from the anode side. Electrochemical impedance spectroscopy was performed in a frequency range of 100 kHz to 100 mHz at a constant voltage.

To test the durability of AEM electrolysis with the X-37-50 T membrane and the NiFe_2_O_4_ catalyst, the test was carried out for 12 h at a current density of 400 mA cm^−2^. Equation (1) was used to calculate the efficiency of the electrolyzer. Faradaic efficiency was also calculated by quantifying the amount of hydrogen produced during electrolysis, where ΔH is the enthalpy of water splitting.

(1) $$Cell\;efficiency(\%)=\frac{moles\;of\;hydrogen\times \Delta H\times 100}{AEM\;electrolyzer\;power\;input}$$

## 4. Conclusions

This study has demonstrated the effectiveness of NiFeO_x_ and NiFe_2_O_4_ catalysts for AEM water electrolysis. NiFe_2_O_4_ displayed the highest catalytic activity for the OER, achieving a current density of 320 mA cm^−2^ at 2.0 V. It also had the lowest Tafel slope at 124 mV/dec, indicating that it possessed the most efficient electrochemical reaction kinetics. Low overpotentials of 416 and 409 mV were achieved for NiFe_2_O_4_ and NiFeO_x_ (5%), respectively, at 10 mA cm^−2^. NiFe_2_O_4_ maintained stability and catalytic activity during the 600 h OER test at a high current density of 50 mA/cm^2^, and over the stability testing period, the degradation rate was 15 μV/h.

NiFe_2_O_4_ demonstrated the highest activity during AEM water electrolysis, with a current density of 1100 mA cm^−2^ at 2.2 V and 45 °C. Current density increased with the increase in electrolysis temperature to 55 °C, and all catalysts exhibited an increase in current density, with the NiFe_2_O_4_ catalyst achieving the highest current density of 1503 mA cm^−2^. Several Fumatech membranes were tested for AEM water electrolysis, including FAA-3-50, FAS-50, FAA-3-PK-75, and FAB PK-130. It was shown that the activity and effectiveness of these membranes are strongly correlated with their ionic conductivity. The most effective membranes were FAA-3-50 and FAS-50, which had comparable performance, whereas FAB PK-130 had the lowest activity compared to the other membranes.

Electrochemical impedance spectroscopy and equivalent circuit fitting analysis were performed on an AEM electrolyzer to examine the impact of various operating parameters. By increasing the temperature from 40 °C to 55 °C, the ohmic resistance reduces. The observed decrease in activation resistance at both the anode and cathode, coupled with improved OH^−^ ion conductivity, signifies faster reaction kinetics and lowered kinetic barriers. These findings highlight the positive impact of temperature elevation on AEM electrolysis. EIS results at 55 °C and 1.7 V for various membrane electrode assemblies highlight distinct performance characteristics. FAA-3-50 and FAS-50 membranes showed lower activation and ohmic resistances, indicative of higher conductivity and faster membrane charge transfer. FAA-3-PK-75 exhibited intermediate performance, while FAB-PK-130 demonstrated poor AEM electrolysis performance, with significantly higher resistances.

The 12 h durability test for the AEM electrolyzers demonstrated robust stability, with a minimal degradation rate of 0.833 mV/h. Throughout the test, the initial voltage of 1.71 V only increased marginally to 1.72 V. The energy efficiency, evaluated at 400 mA/cm^2^, was 72%, indicating efficient hydrogen production during the entire duration of the durability test.

## Figures and Tables

**Table 1:** Specifications of Fumasep membranes [42,43,45,46].

Membrane	Thickness ( μm)	Specific Conductivity in Cl^−^ (mS cm^−1^)	Specific Area Resistance (Ω cm^−2^)
FAA-3-50	45–55	3–8	0.6–1.5
FAS-50	45–55	3–8	0.6–1.5
FAA-3-PK-75	70–80	4.5–6.5	1.2–2
FAB PK-130	110–140	1–2.5	5–9

**Table 2:** Results from fitting an equivalent circuit during electrolysis operations at temperatures of 45 °C and 55 °C using FAA-3-50 membrane, with NiFe_2_O_4_ catalyst.

Temperature	Ohmic Resistance	Activation Resistance
	R_ohm_ (Ω)	R_anode_ + R_cathode_ (Ω)
1.7 V		
45 °C	0.127	0.101
55 °C	0.108	0.075
1.8 V		
45 °C	0.133	0.077
55 °C	0.093	0.071
1.9 V		
45 °C	0.134	0.051
55 °C	0.116	0.036
2 V		
45 °C	0.134	0.055
55 °C	0.117	0.031

**Table 3:** Resistances evaluated from equivalent circuit fittings for AEM water electrolysis using different membranes with NiFe_2_O_4_ catalyst at 1.7 V.

Membranes	Ohmic Resistance	Activation Resistance
	R_ohm_ (Ω)	R_anode_ + R_cathode_ (Ω)
FAA-3-50	0.108	0.0756
FAS-50	0.092	0.0918
FAA-3-PK-75	0.16	0.113
FAB-PK-130	0.777	0.199

## Data Availability

Data are contained within the article and Supplementary Materials.

## Supplementary Materials

The following supporting information can be downloaded at: https://www.mdpi.com/article/10.3390/molecules30153228/s1.
