# Supplementary material for: Enhanced NiFe2O4 Catalyst Performance and Stability in Anion Exchange Membrane Water Electrolysis: Influence of Iron Content and Membrane Selection
Source: Molecules. 2025 Aug 1;30(15):3228. doi: 10.3390/molecules30153228 (PMC12348109; doi:10.3390/molecules30153228)
Supplement: Supplementary file 1 [file molecules-30-03228-s001.zip › molecules-3750513-supplementary.pdf]

Supplementary Information

Enhanced $NiFe_2O_4$ Catalyst Performance and Stability in Anion Exchange Membrane Water Electrolysis: Influence of Iron Content and Membrane Selection.

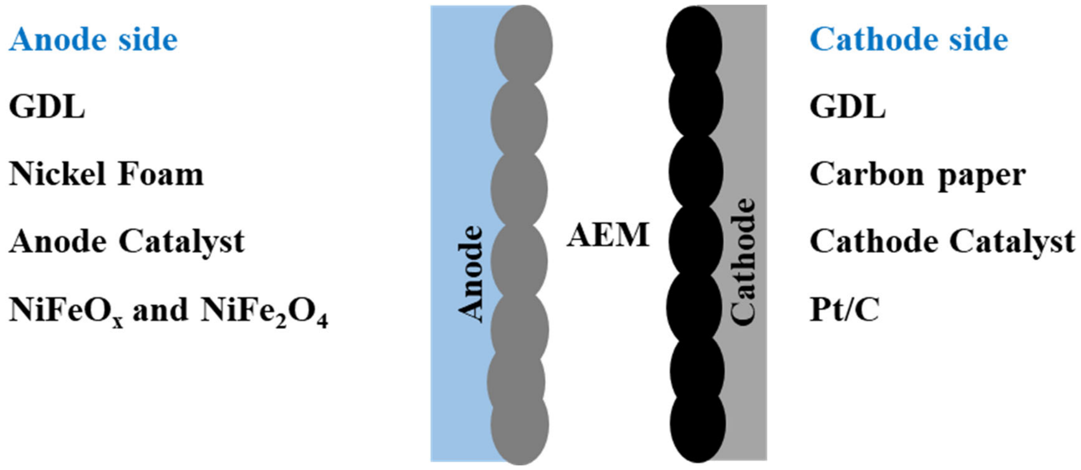

Figure S1. Schematic of membrane electrode assembly used in this study.

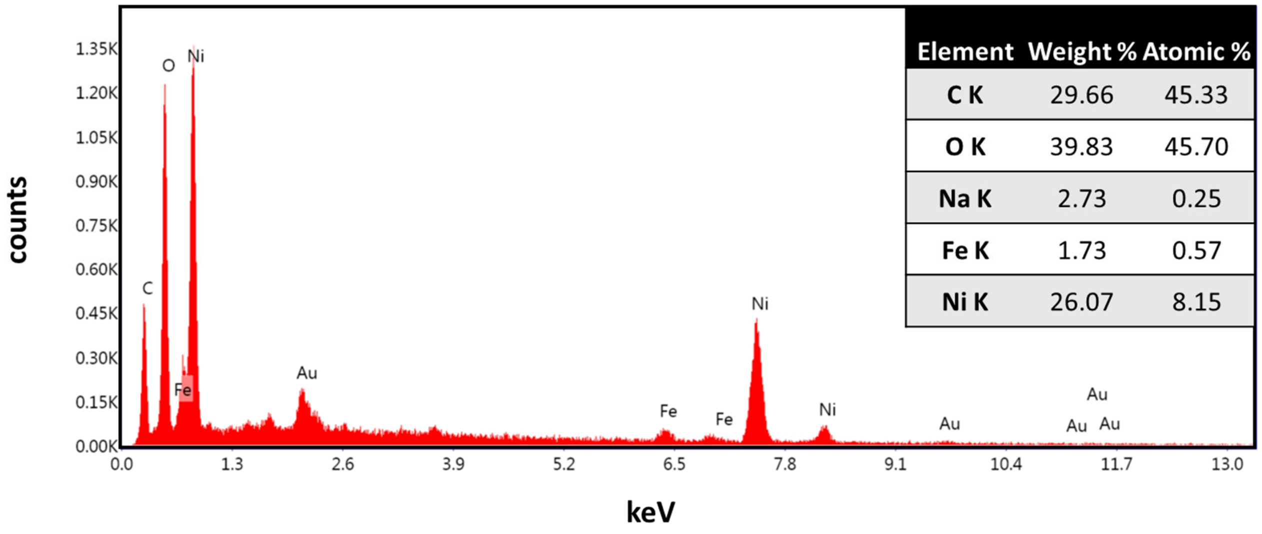

Element	Weight %	Atomic %
C K	29.66	45.33
O K	39.83	45.70
Na K	2.73	0.25
Fe K	1.73	0.57
Ni K	26.07	8.15

Figure S2. EDS spectra of $NiFeO_x$ (5% Fe).

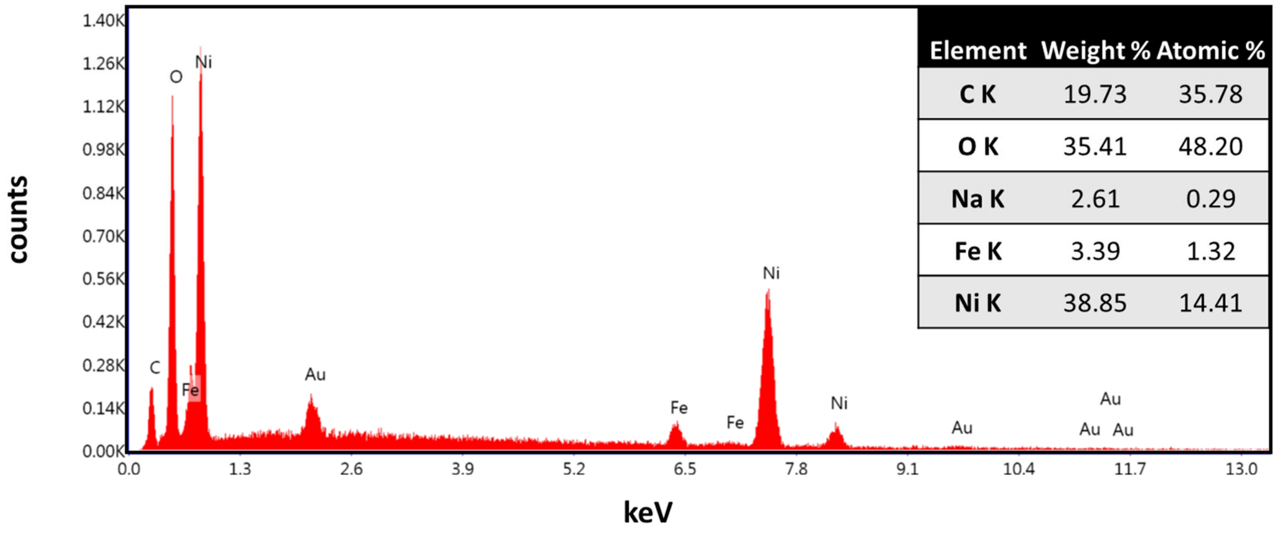

Element	Weight %	Atomic %
C K	19.73	35.78
O K	35.41	48.20
Na K	2.61	0.29
Fe K	3.39	1.32
Ni K	38.85	14.41

Figure S3. EDS spectra of $NiFeO_x$ (7.5% Fe).

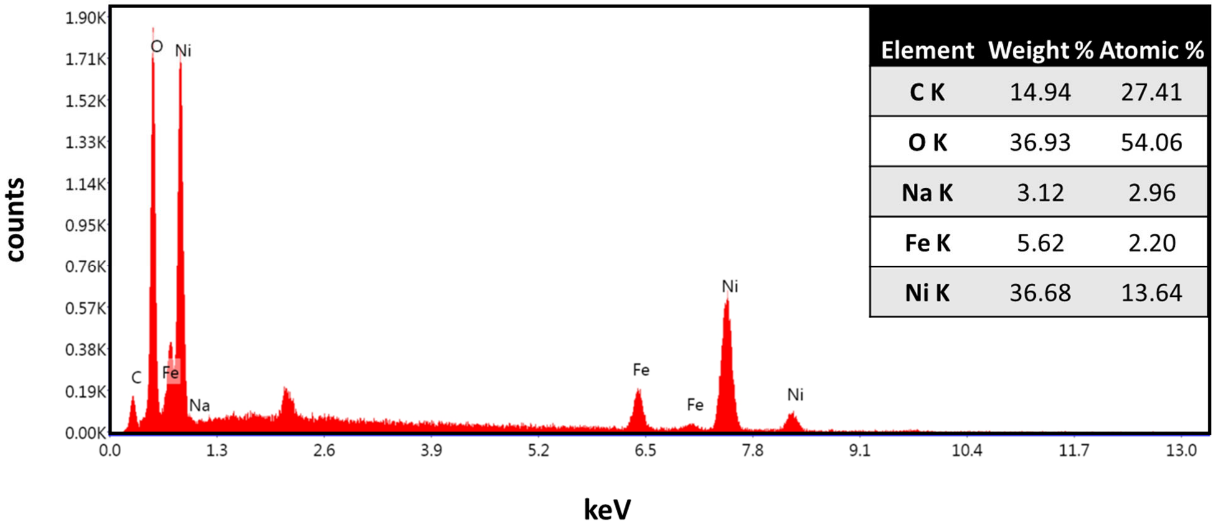

Figure S4. EDS spectra of $NiFeO_x$ (12.5% Fe).

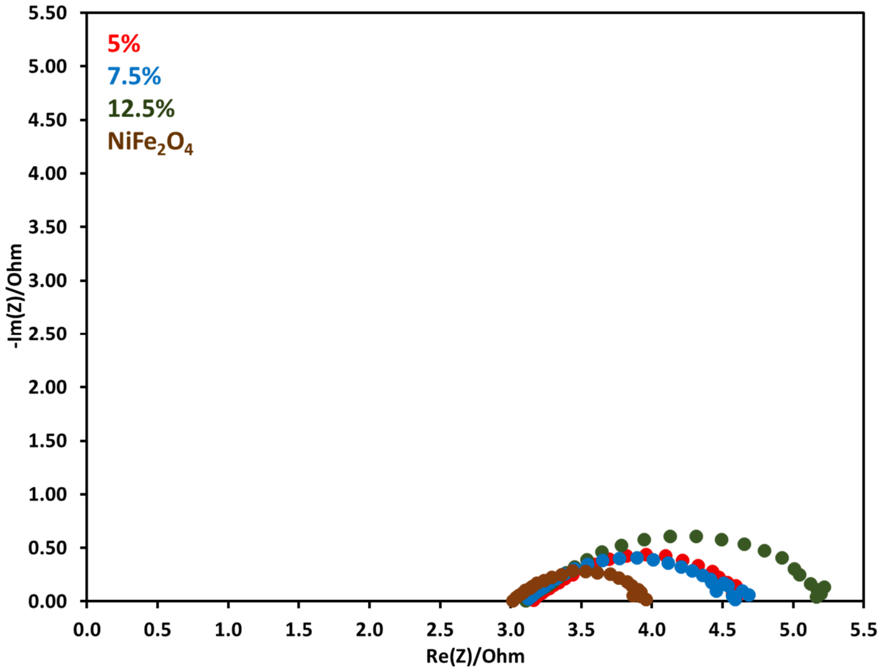

Figure S5. Nyquist plot for OER at 25°C using 1M KOH with $NiFeO_x$ and $NiFe_2O_4$ catalysts.
